# The effect of the therapy of “combination 3 methods progression” in patients with neurogenic bowel dysfunction (constipated type): Study protocol for a randomized controlled trial

**DOI:** 10.1097/MD.0000000000024662

**Published:** 2021-02-19

**Authors:** Qing Li, Yin-Li Shen, Yun-Lan Jiang, Dong-Shuang Li, Song Jin

**Affiliations:** a Nursing School, Chengdu University of Traditional Chinese Medicine; b Department of Rehabilitation, Affiliated Hospital of Chengdu University of Traditional Chinese Medicine; c College of Clinical Medicine, Chengdu University of Traditional Chinese Medicine, Chengdu, Sichuan Province, PR China.

**Keywords:** combination 3 methods progression, constipation, neurogenic bowel dysfunction, protocol, randomized controlled trial

## Abstract

**Background:**

As one of the important manifestations of neurogenic bowel dysfunction, constipation is characterized by high incidence and harmful effects. It has a negative impact on both physical and psychological health of patients. And there are no effective treatment options for this type of disease clinically. Therefore, this study is designed to examine the effect of the therapy of “combination 3 methods progression” in patients with neurogenic bowel dysfunction (constipated type).

**Methods:**

This is a randomized, controlled, parallel-design clinical trial. A total of 60 patients with neurogenic bowel dysfunction (constipated type) will be randomly assigned to intervention group and control group. The control group will receive 4 weeks of usual rehabilitation care, the intervention group will receive 4 weeks of the therapy of “combination 3 methods progression” in addition to usual rehabilitation care. The primary outcome is the number of spontaneous bowel movement per week. Secondary outcomes are stool characteristics, degree of difficulty in defecation, level of anxiety, level of depression, and level of self-efficacy.

**Discussion:**

The interventions of this protocol have been programmed to alleviate constipation in patients with neurogenic bowel dysfunction. Findings may provide preliminary evidence for clinical efficacy of the therapy of “combination 3 methods progression.”

**Trial registration:**

Chinese Clinical Trial Registry, IDF: ChiCTR2000041463. Registered on December 26, 2020.

## 1 Introduction

### 1.1 Background

Neurogenic bowel dysfunction (NBD) is a disorder of bowel dysfunction caused by neurological deficits (including nerve damage, neurological disease, and congenital defects in the nervous system).^[1,2]^ The main clinical manifestations are constipation, fecal incontinence, abdominal pain, and distention.^[3–5]^ Spinal cord injury (SCI) and stroke are among the common primary diseases of NBD.^[6]^ According to the epidemiological data, about 56% to 95% of SCI patients have constipation,^[3,7]^ with 30% of them being severely constipated.^[8]^ And the prevalence of constipation in stroke patients varies from 30% to 66%.^[9–11]^ The pathogenesis of NBD is complex and varies with the location and severity of neuropathy,^[12]^ but it is very certain that it not only causes harm to physical and psychological health, but also causes patients to receive serious effects on daily life and social relationships.^[13–15]^ Therefore, bowel management is very necessary for NBD patients.

Currently, treatments for NBD vary and mainly include dietary management,^[1,16]^ medication (e.g., laxatives, suppositories),^[17,18]^ frequency electric stimulation,^[19]^ finger stimulation,^[20,21]^ and transanal irrigation.^[22]^ In addition, traditional Chinese medicine (TCM) therapies, such as acupuncture and moxibustion,^[23]^ electroacupuncture,^[24,25]^ and scrapping therapy,^[26]^ and so on, are also slightly effective in the treatment of NBD. Unfortunately, many of them are time-consuming and costly, with poor compliance and limited effectiveness. Thus, there is a need for a safer, more convenient, and effective therapy to manage the bowel.

The therapy of “combination 3 methods progression” is a rehabilitation program that combines tradition Chinese medicine and Western medicine. It includes 3 aspects: pestle needle therapy, Tuina, and functional exercise. Pestle needle therapy was developed in the 1970s by Zhong-Yu Li, a famous professor in Chengdu University of TCM.^[27]^ It is an important branch of acupuncture in tradition Chinese medicine. It stimulates the acupoints on the body surface through certain techniques. The needles do not pierce the skin, but only act on the internal organs and meridians, thus achieving the effect of treating diseases and strengthening body.^[28,29]^ It has the advantages of precise acupoints, painless, noninvasive, easy to operate, and widely applicable to the population.^[30,31]^ Pestle needle has the dual effect of acupuncture and massage,^[32,33]^ and has been widely used in the treatment of various diseases.^[34]^ Tuina is a traditional form of manipulative therapy, and is essentially a Chinese medical massage.^[35,36]^ The therapeutic effect is achieved by applying a certain force to the muscles or soft tissues of the body through the operator's fingers, hands, elbows, knees, and feet.^[37]^ Tuina has been shown to have a positive impact in the treatment of constipation.^[38]^ Functional exercise is a common form of rehabilitation therapy in Western medicine. A few studies have shown that functional exercise can improve the physical and mental health of people with constipation, and its safety has been proven.^[39–41]^ In our previous studies, we also found that constipation symptom in NBD patients was reduced by instructing them to perform functional exercises, but it needs further confirmation. The therapy of “combination 3 methods progression” was developed by professor Song Jin on the basis of long-term clinical practice and experience in disease rehabilitation. It emphasizes the combination of TCM and modern rehabilitation techniques. In past clinical studies, this therapy has been shown to be effective in chronic diseases.^[42]^ At present, there are no clinical studies on the application of it to NBD patients. Based on these uncertainties, the aim of our study is to determine whether “combination 3 methods progression” is effective in patients with NBD.

### 1.2 Objectives

In this study, the first objective is to investigate the clinical efficacy of “combination 3 methods progression” in the treatment of constipation in NBD patients. The second objective is to determine whether the therapy can reduce anxiety and depression in NBD patients. The third objective is to explore a more scientific approach to bowel management for NBD patients.

### 1.3 Trial design

**Figure 1:**
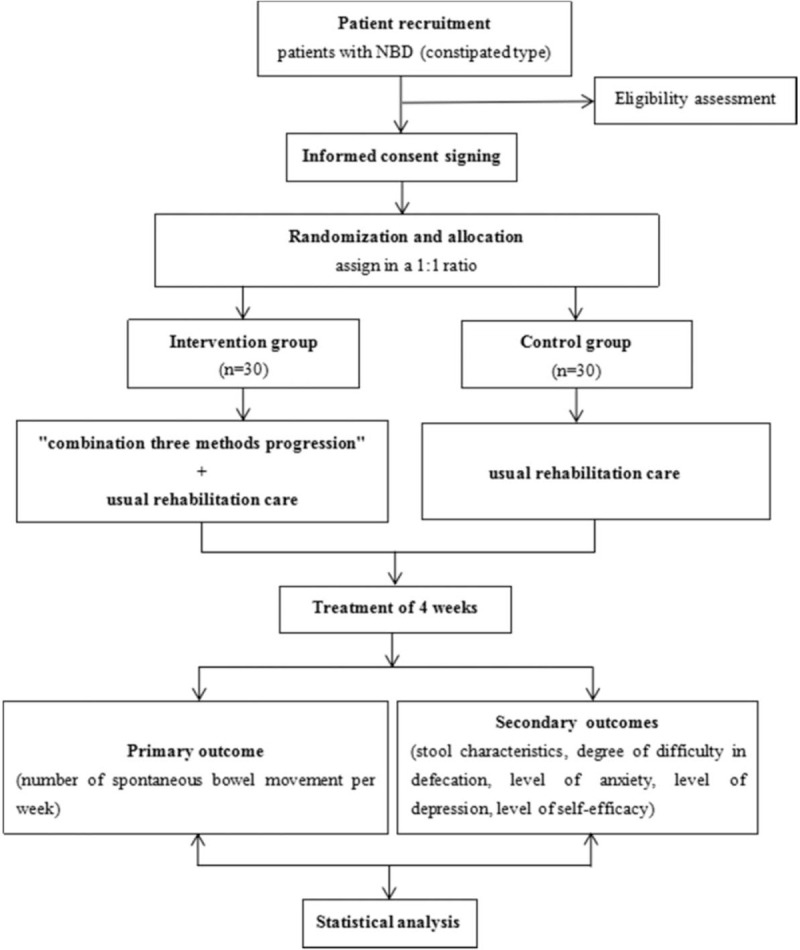
Trial flow chart.

This is a randomized, controlled, parallel-design clinical trial which will be implemented from March 2021 to March 2022. The study follows the Declaration of Helsinki Principles.^[43]^ It was allowed by Medical Ethics Committee of Affiliated Hospital of Chengdu University of TCM (2020KL-076), and has been registered on the Chinese Clinical Trial Registry (Registration No. ChiCTR2000041463). The protocol will be reported according to Standard Protocol Items: Recommendations for Interventional Trials statement. The flowchart of the trial is shown in Figure 1.

## 2 Methods

### 2.1 Study setting, recruitment, and informed consent

The trial will be carried out at the Affiliated Hospital of Chengdu University of TCM. Sixty patients will be recruited by posting recruitment ads on hospital outpatient clinics and hospital ward bulletin boards. A contact number will be included in the recruitment information so that potential participants can contact us. The study will be available to all NBD patients (constipated type) interested in the therapy of “combination 3 methods progression.” Two trained investigators will describe the study to the patients (if applicable), including the purpose, content, duration, and frequency of interventions, benefits, possible discomfort and risks, and associated costs. All patients who agree to participate in this study will sign an informed consent form.

### 2.2 Inclusion criteria

(1) Meet the international standard for neurological classification of SCI developed by American Spinal Injury Association: Grades B to D, confirmed by computed tomography (CT) or magnetic resonance imaging (MRI)^[44]^; or meet the diagnostic criteria for cerebral hemorrhage,^[45]^ confirmed by CT or MRI; or meet the diagnostic criteria for cerebral infarction,^[46]^ confirmed by CT or MRI;
(2) Meet the Roman IV diagnostic criteria for functional constipation^[47]^;
(3) With symptoms of anxiety and depression;
(4) Aged 20 to 80 years;
(5) With stable vital signs, clear consciousness, normal communication, and expression skills;
(6) Sign an informed consent form and submit to the arrangements of the subject group.

Note: Only patients who meet the above 6 criteria can be included in this study.

### 2.3 Exclusion criteria

(1) With serious primary diseases of the heart, liver, kidney, and endocrine system;
(2) Pregnant and lactating patients;
(3) With severe clotting disorders;
(4) With psychiatric disorders;
(5) Skin allergies or breakouts in the treated area;
(6) Already participated in other trials.

Note: Patients who meet any of the above criteria should be excluded in this study.

### 2.4 Randomization and blinding

Randomization will be based on a randomization sequence generated by the software SPSS25.0. Patients will be randomized at a 1:1 ratio into intervention group and control group. Randomization will be performed by a statistician who is not involved in this study. Data managers, and outcome assessors will be unaware of the outcome. However, due to the trail's nature, the doctors, nurses, and therapists will not be blinded.

### 2.5 Interventions

#### 2.5.1 Intervention description

##### 2.5.1.1 Control group

Receiving usual rehabilitation care, the duration is 4 weeks.

(1) Dietary care: nurses will instruct patients to eat more fruits, vegetables, coarse grains, and other high-fibre and nutrient-rich foods, avoid beans and milk, and ensure adequate daily intake of water to soften the stools, thus providing a smooth bowel movement.
(2) Defecation care: use suppositories or laxatives as an aid to defecation as prescribed by the doctor. If the patient is severely constipated, perform a manual cleansing enema for defecation.
(3) Psychological care: ① Nurses will communicate with patients in a cordial and amiable manner in order to establish a good nurse-patient relationship. ② Actively explain to patients about constipation to increase their knowledge of this disease. ③ Take the initiative to communicate with the patients’ family to increase social support and patients’ confidence. ④ Encourage patients to divert negative emotions by listening to music and chatting, and so on.

##### 2.5.1.2 Intervention group

Receiving the therapy of “combination 3 methods progression” in addition to usual rehabilitation care.

###### 2.5.1.2.1 Pestle needle treatment

**Figure 2:**
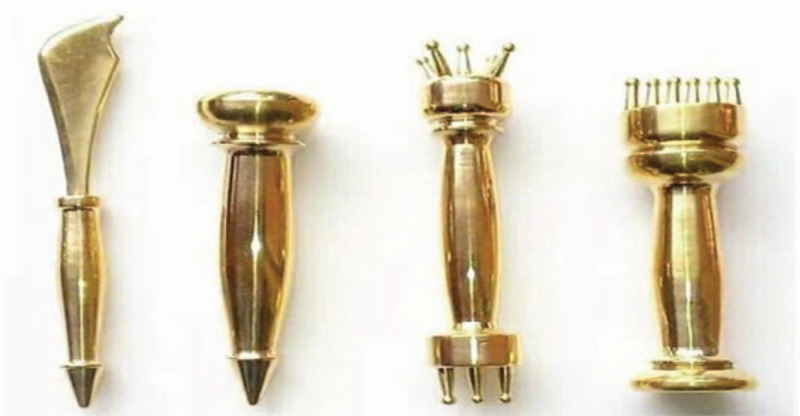
Diagram of pestle needle (from left to right: Kui Xing Pen, Vajra Pestle, Five star-Three terrace pestle, Seven Luminaries-mixed Yuan Pestle).

**Figure 3:**
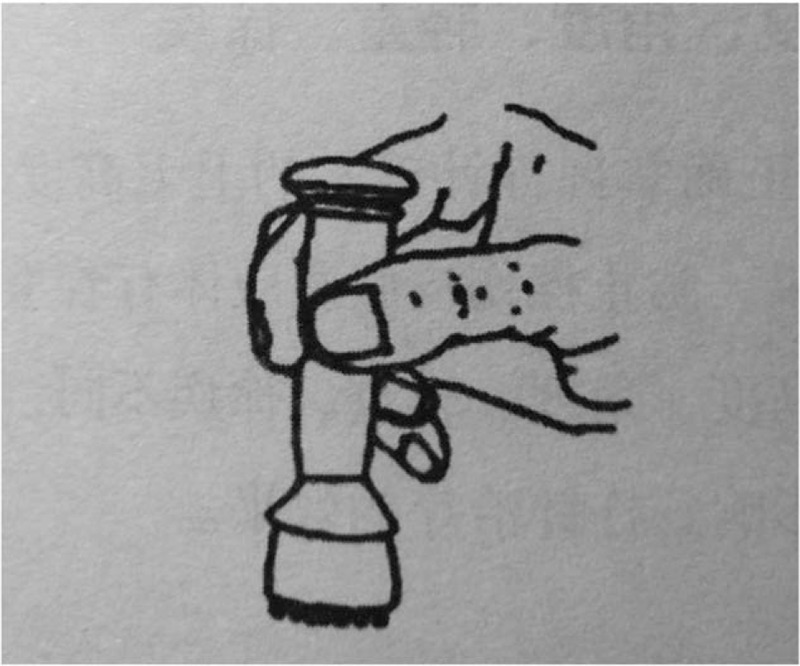
Pencil operated method.

**Figure 4:**
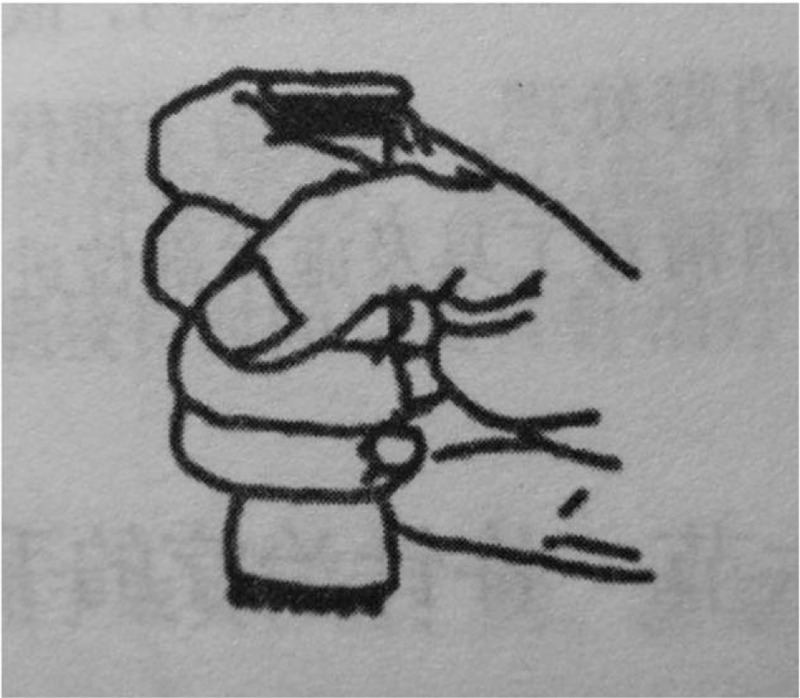
Straight grip method.

**Figure 5:**
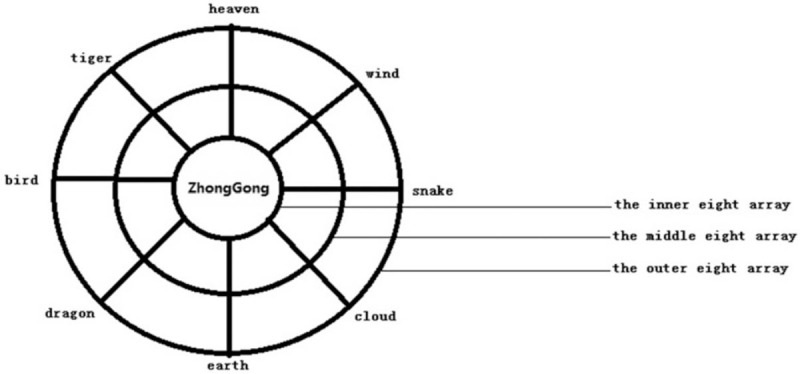
Diagram of Bazheng acupoints.

**Figure 6:**
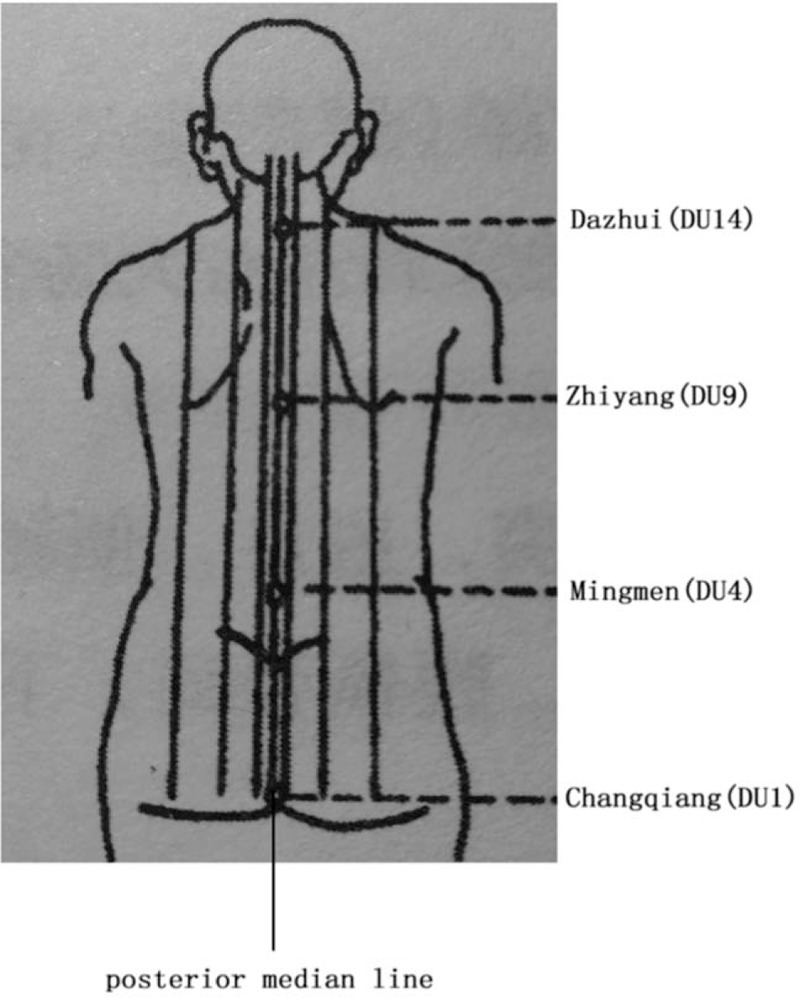
Diagram of Heche road.

The tool used in this treatment is Taiji pestle needle, which was designed by Li's pestle needle heritage workshop of Affiliated Hospital of Chengdu University of TCM. It includes 4 different types of needles: Seven luminaries-mixed Yuan Pestle, Five Star-Three Terrace Pestle, Vajra Pestle, and Kui Xing Pen (Fig. 2). They are all in full brass and their lengths are 10.5 cm, 11.5 cm, 10.5 cm, and 8 cm respectively. The method of holding pestle needle includes pencil operated method (Fig. 3) and straight grip method (Fig. 4). Pencil operated method is suitable for the treatment of head, face, chest, abdomen, and areas with few muscles of extremities. Straight grip method is suitable for the treatment of waist, back, sacrum, and areas with rich muscles of extremities. Treatment techniques include Diankou, Shengjiang, Kaihe, Yunzhuan, and Fenli (Table 1). The criteria for the selection of acupoints refer to the book “Pestle Needle Studies” published by the Chinese Press of Traditional Chinese Medicine.^[48]^ There are 2 specific acupoints: Bazheng acupoints (8 array acupoints) and Heche Road (the road for the vehicle). Using an acupoint as Zhong Gong, a circle is drawn with a certain distance (usually is 3 cun, and 1 cun≈20 mm) as a radius from the center of Zhong Gong. This circle is then divided into 8 equal parts (heaven, earth, wind, cloud, dragon, tiger, bird, and snake) to form 8 acupoints, and is called the outer 8 array. The distance from Zhong Gong to the outer 8 array is then divided into 3 equal parts and 2 circles are drawn, which are called the middle and inner 8 array. The acupoints on the inner, middle, and outer 8 array form the Bazheng acupoints (Fig. 5). The back of the human body has 7 Heche Road (Fig. 6), one of which is the posterior median line and the other 6 are parallel lines of 0.5 cun, 1.5 cun, and 3 cun apart from the posterior median line. The number of acupoints selected for this therapy is 13 (Table 2). The operation steps are as follows: ① Take the 8 array acupoints of Shendao (DU11), use Vajra Pestle and Seven Luminaries-Mixed Yuan Pestle to perform Fenli technique for about 3 minutes, then use the tip of Five Star-Three Terrace Pestle to perform Diankou technique for about 3 minutes. ② Take the 8 array acupoints of Yaoyangguan (DU3) and perform with the same tools and techniques as above. ③Take the Heche Road of Da zhui (DU14) to Changqiang (DU1), use the tip of the Seven Luminaries-Mixed Yuan Pestle to perform Shengjiang technique for about 5 minutes or until skin is slightly red. Then use the tip of Kui Xing Pen to perform Kaihe technique and the tip of Five Star-Three Terrace Pestle to perform Diankou technique for about 3 minutes respectively. Finally, use the shank of Vajra Pestle to perform Yunzhuan technique for about 1 minute. ④ For those who suffer from emotional discomfort and sadness, add Zhangmen (LR13), Sanyinjiao (SP6), and Taichong (LR3). For those who have weak bowel movements, add Tianshu (ST25), Zushanli (ST36), and Shangjuxu (ST37). For those who like to eat hot and spicy food, and drink insufficient water, add Xuehai (SP10), Weizhong (BL40), Chengshan (B57), Zushanli (ST36), Sanyinjiao (SP6), and Baliao acupoint. The frequency of treatment is about once a day, 5 days a week and the duration is 4 weeks.

**Table 1:** The detailed methods of treatment techniques for pestle needle therapy.

Treatment techniques	Detailed methods
Diankou	The tip of pestle needle is tapped repeatedly on the acupoints, like a sparrow pecking. It is suitable for acupoints with small areas.
Shengjiang	Use the tip of pestle needle to touch the skin at acupoints, then move up and down along the longitudinal axis of body, move up is named “sheng” and move down is named “jiang.” It is suitable for acupoints with large areas.
Kaihe	Use the tip of pestle needle to touch the skin at acupoints, and apply force in a vertical direction, as much as the patients can tolerate, it is named “kai.” Then stop applying force and lift pestle needle slowly upwards, without the tip leaving the skin of acupoints, it is named “he.” It is suitable for acupoints with small areas.
Yunzhuan	Use the tip of Seven luminaries-mixed Yuan Pestle and Five star-Three Terrace Pestle, or use the shank of Vajra Pestle and Kui Xing Pen firmly against the skin at acupoints, and move it in a clockwise or counterclockwise direction.
Fenli	Use the tip or the shank of pestle needle to touch the skin at acupoints, move from side to side along the horizontal axis of the body, it is named “fen.” Then move up and down along the longitudinal axis of the body, it is named “li.” It is suitable for acupoints with large areas.

**Table 2:** Location of acupoints for pestle needle therapy.

Acupoint	Location
Eight array acupoints of Shendao (DU11)	Using Shendao (DU11) as Zhong Gong (DU11 is located in the depression below the spinous process of the 5th thoracic vertebra), the distance from Shendao (DU11) to Shentang (BL44) is the radius.
Eight array acupoints of Yaoyangguan (DU3)	Using Yaoyangguan (DU3) as Zhong Gong (DU3 is located in the depression below the spinous process of the 4th lumbar vertebra), the distance from Yaoyangguan (DU3) to Dachangshu (BL25) is the radius.
Heche Road of Da zhui (DU14) to Changqiang (DU1)	The posterior median line from Da zhui (DU14) to Changqiang (DU1) and 3 parallel lines on each side of the posterior median line, 7 lines in total.
Zhangmen (LR13)	In the mid-axillary line, below the end of the 11th rib.
Sanyinjiao (SP6)	On the inner side of calf, 3 cun above the tip of inner ankle, at the posterior border of the medial edge of tibia.
Taichong (LR3)	Dorsal side of foot, posterior depression of the 1st metatarsal space.
Tianshu (ST25)	Two cun next to the anterior median line, level with the middle of navel.
Zusanli (ST36)	On the outer side of the lower leg, 3 cun below Dubi (ST35).
Shangjuxu (ST37)	On the outer side of the lower leg, 6 cun below Dubi (ST35).
Xuehai (SP10)	Bend the knee, on the inner thigh, 2 cun above the medial end of patellar base, at the bulge of the medial head of quadriceps.
Weizhong (BL40)	Midpoint of the transverse popliteal line.
Chengshan (BL57)	In the middle of the back of calf, between Weizhong (BL40) and Kunlun (BL60), in the depression under the belly of the gastrocnemius muscle when the calf is straightened and the heel is lifted.
Baliao acupoint	①Shangliao (BL31): In the sacral region, between the posterior superior iliac spine and the posterior median line, at the first posterior sacral foramen. ②Ciliao (BL32): In the sacral region, below the internal posterior superior iliac spine, at the second posterior sacral foramen. ③Zhongliao (BL33): In the sacral region, medial to Ciliao (BL32), at the third posterior sacral foramen. ④Xialiao (BL34): In the sacral region, below the inner part of Zhongliao (BL33), at the fourth posterior sacral foramen.

###### 2.5.1.2.2 Tuina treatment

Treatment techniques include Zhian, Moyun, Zhirou, and Tinie (Table 3). The criteria for the selection of acupoints refer to the book “Tuina Studies” published by the Chinese Press of Traditional Chinese Medicine.^[49]^ The number of acupoints selected for this therapy is 12 (Table 4). The operation steps are as follows: ① The patient is placed in a supine position and Zhirou technique is applied to Zhongwan (RN12), Guanyuan (RN4), Qihai (RN6), Tianshu (ST25), Daheng (SP15) in turn. ② Place the patient in a prone position, apply Tinie technique to Changqiang (DU1) and Zhirou technique to Pishu (BL20), Weishu (BL21), Ganshu (BL18), Dachangshu (BL25). ③ Apply Zhian technique to Shenshu (BL23), Changqiang (DU1), and Moyun technique to Baliao acupoint. The frequency of treatment is about 20 minutes once a day, 5 days a week and the duration is 4 weeks.

**Table 3:** The detailed methods of treatment techniques for Tuina.

Treatment techniques	Detailed methods
Zhian	Use the end of thumb to apply vertical pressure to the body surface. When a single finger is not strong enough, the thumb of the other hand can be overlapped to complement the pressure. It is often used in combination with Zhirou.
Moyun	Gentle rubbing movements with fingers, palm roots, thenar, or hypothenar muscles on the surface of body, either in a centrifugal or centripetal direction.
Zhirou	Apply the abdomen of fingers (thumb, middle finger, index finger, middle finger, or ring finger) to a certain area or acupoint and rub it in a gentle, rhythmic motion.
Tinie	Use thumb and other 4 fingers to make a squeezing motion on the surface of body.

**Table 4:** Location of acupoints for Tuina.

Acupoint	Location
Zhongwan (RN12)	On the anterior median line of body, 4 cun above the navel.
Guanyuan (RN4)	On the anterior median line of body, 3 cun below the navel.
Qihai (RN6)	On the anterior median line of body, 1.5 cun below the navel.
Tianshu (ST25)	Refer to Table 2.
Daheng (SP15)	Four cun next to the anterior median line of body, level with the middle of the navel.
Pishu (BL20)	In the back, 1.5 cun next to inferior end of the spinous process of the 11th thoracic vertebra.
Weishu (BL21)	In the back, 1.5 cun next to inferior end of the spinous process of the 12th thoracic vertebra.
Ganshu (BL18)	In the back, 1.5 cun next to inferior end of the spinous process of the 9th thoracic vertebra.
Dachangshu (BL25)	In the back, 1.5 cun next to inferior end of the spinous process of the 4th lumbar vertebra.
Shenshu (BL23)	In the back, 1.5 cun next to inferior end of the spinous process of the 2th lumbar vertebra.
Changqiang (DU1)	In the depression below the tip of tailbone.
Baliao acupoint	Refer to Table 2.

###### 2.5.1.2.3 Functional exercise

① Explain the importance of functional exercise and instruct the patient to perform abdominal breathing for 40 times. ② Instruct the patient to massage abdomen clockwise with the base of palm until skin is slightly red. ③ Instruct the patient to perform straight leg raising for 15 to 20 times, bridge-style movement for 5 to 10 times and hip contracting exercise for 10 to 15 times, then relaxing after lasting 5 seconds each time. ④ Pull the anal sphincter horizontally and vertically for 10 to 15 times respectively. ⑤ Finger stimulation for 15 to 20 seconds until the bowel wall is relaxed and feces flows. ⑥ Instruct the patient to simulate defecation. The frequency of treatment was once a day, 5 days a week and the duration is 4 weeks.

#### 2.5.2 Criteria for discontinuing or modifying allocated interventions

If the patient suddenly develops other serious illnesses or requests to withdraw from the trial voluntarily, the interventions will be discontinued.

#### 2.5.3 Strategies to improve adherence to interventions

All treatment and care measures will be carried out by clinically experienced doctors, therapists, and nurses. Before the trial begins, they will receive training to ensure that all interventions are completed with high qualities.

### 2.6 Outcomes

#### 2.6.1 Plans for assessment and collection of outcomes

Demographic and baseline data will be measured by questionnaires before the interventions. The primary outcome and secondary outcomes will be measured by checklists or scales. They will be assessed before the interventions, after 2 weeks of interventions and after 4 weeks of interventions.

#### 2.6.2 Primary outcome

The primary outcome is the number of spontaneous bowel movement per week. It is defined as the number of spontaneous and complete bowel movement without the need of medication or manipulation. The trial investigator will use diary cards to count the number of spontaneous bowel movement in a week.

#### 2.6.3 Secondary outcomes

(1) Stool characteristics: it will be evaluated with Bristol stool form scale,^[50]^ which is scored by typology. One point means type 1, 2 points means type 2, and so on. Type 1 is separate hard lumps, like nuts (hard to pass); type 2 is sausage-shaped but lumpy; type 3 is like a sausage, but with cracks on surface; type 4 is like a sausage or snake, smooth, and soft; type 5 is soft blobs with clear-cut edges (passed easily); type 6 is fluffy pieces with ragged edges, a mushy stool; and type 7 is watery, no solid pieces.
(2) Degree of difficulty in defecation: it will be evaluated with scoring method by checklists. One point means no difficulty; 2 points means that force is required to pass the stool; 3 points means very strong force is required to pass the stool; 4 points means the aid of massage around the belly or even hand picking are required to pass the stool.^[51]^
(3) Level of anxiety: it will be evaluated with anxiety self-rating scale.^[52]^ The scale include 20 items and adopts 4-grade scoring method, 1 point means no or very little time, 2 points means little time, 3 points means a lot of time and 4 points means most or all of the time. Calculation method: crude score is calculated first, then crude score is multiplied by 1.25 to obtain standard score, which is >50 as having anxiety symptoms, the higher the score, the more severe the symptoms.
(4) Level of depression: it will be evaluated with self-rating depression scale.^[53]^ It has the same number of items, scoring method, and calculation method as self-rating scale.
(5) Level of self-efficacy: it will be evaluated with general self-efficacy scale.^[54]^ The scale include 10 items and adopt 4-grade scoring method, 1 point means not at all correct, 2 points means somewhat correct, 3 points means mostly correct, 4 points means completely correct. Calculation method: total score is 10 to 40, with 0 to 19 being low, 20 to 29 being medium, and 30 to 40 being high. Higher scores indicate a stronger sense of self-efficacy.

### 2.7 Data management and confidentiality

All patients will be numbered according to the order of admission time and randomly grouped. To avoid the occurrence of measurement bias, relevant data were collected by uniformly trained individuals who were not involved in the intervention process and were not aware of the groups. All data obtained from NBD patients will be recorded on case record form and entered into the software SPSS25.0. Two different individuals will enter data to ensure the accuracy. The obtained data, including checklists, scales, questionnaires, and informed consent forms will be stored in locked file cabinets, and will be kept strictly confidential. Only researchers will have access to the participants’ data in the study.

### 2.8 Sample size

In this study, the following formula was selected to estimate the sample size:

$$n=\frac{2{\left(\mu_{\alpha }+\mu_{\beta }\right)}^{2}P(1-P)}{\delta^{2}},P=(P_{1}+P_{2})/2$$

*P*_1_ is the effective rate of intervention group, *P*_2_ is the effective rate of control group. Based on pre-experimental results, *P*_1_ = 85%, *P*_2_ = 50%. According to statistical requirements, the *α* was set to 5%, and the *β* was set to 80%. By looking up the table of random numbers, we get $\mu_{\alpha }$ at 1.96, $\mu_{\beta }$ at 0.84, the estimated sample size was 28 patients per arm. To ensure the replenishment of lost samples, the sample content is increased by 20%, so the final sample size was 30 patients per arm.

### 2.9 Statistical analysis

The software SPSS25.0 was used for statistical analysis of data. The statistician will be blinded to the whole trial process. The continuous variables will be showed as the mean ± standard deviation and performed with a paired samples *t*-test or independent *t*-test. The categorical variables will be presented as percentage (%), and performed with Pearson Chi-squared test. For all comparisons, if a *P*-value is less than .05, the difference is considered statistically significant.

### 2.10 Plans to give access to the full protocol, participant-level data, and statistical code

The time of sharing data and the full protocol is within 6 months after completion of the trial. The full protocol will be published in a peer-reviewed journal.

### 2.11 Dissemination plans

We will submit the results of the study to be published in a peer-reviewed journal.

## 3 Discussion

As far as we know, this is the first study to evaluated the effect of “combination 3 methods progression” in treating constipation in NBD patients. As an important part of NBD patients, the health problems of SCI patients and stroke patients have been paid more and more attention. SCI was not only considered to be an incurable condition, but also an inestimable public health issue.^[55–58]^ Several studies have shown that SCI was a disease with a high rate of disability and death.^[59,60]^ In China, the incidence of SCI was approximately 23.7 to 60.0 cases per million population, with a relatively rapid growth trend.^[61]^ In addition, stroke has similar disease characteristics. Fifteen million people worldwide suffer a stroke each year, resulting in 5 million deaths and 5 million permanent disabilities.^[62]^ In low-to-middle-income countries, the incidence of stroke continues to increase, accounting for 85% of the world's stroke burden.^[63]^ Patients with NBD will experience a long recovery period, which places a huge financial burden on their family and society. Therefore, it is vital to identify safe and effective treatment methods to solve constipation problem.

TCM and Western medicine have their own unique advantages in the treatment of chronic diseases. Clinical studies have shown that a single treatment modality for constipation symptom in NBD patients cannot achieve satisfactory results.^[64]^ The therapy of “combination 3 methods progression” combines pestle needle therapy with Tuina and functional exercise to form a unique and comprehensive rehabilitation program. The advantages of the therapy are clear: ①The pestle needle overcomes the disadvantage of traditional acupuncture piercing the skin, avoids patients’ fear and the risk of infection, and greatly improves patients’ compliance. ②Low cost of treatment, reducing financial pressure on patients. ③ The operations are very easy and flexible to perform. ④It has no side effects and is highly safe. Because of this, we have also made some achievements in the previous clinical studies.

In conclusion, we believe that this randomized trial will provide more evidence regarding whether the therapy can reduce constipation, anxiety, and depression in NBD patients.

## Acknowledgment

The authors are grateful to the Sichuan Provincial Administration of Traditional Chinese Medicine for funding this study.

## Author contributions

**Conceptualization:** Yin-Li Shen, Yun-Lan Jiang, Song Jin.

**Data curation:** Yin-Li Shen, Dong-Shuang Li.

**Investigation:** Qing Li, Yin-Li Shen.

**Project administration:** Song Jin.

**Resources:** Yin-Li Shen.

**Software:** Qing Li.

**Writing – original draft:** Qing Li.

**Writing – review & editing:** Yun-Lan Jiang.
